# Hematological profiles of COVID-19 patients at the Ratlam district, Madhya Pradesh State, India

**DOI:** 10.6026/97320630017686

**Published:** 2021-07-31

**Authors:** Reetesh Kumar Gujar, Anil Meena, Shailendra Singh Chouhan, KS Likhar

**Affiliations:** 1 Department of Pathology, Government Medical College Ratlam, Madhya Pradesh 457001, India

**Keywords:** Hematology, NLR, SII, ROC curve, COVID

## Abstract

It is of interest to compare the hematological profile (using Complete blood count) of COVID patients admitted in ICU, private ward, and isolation ward with varying severity. This data will help predict the severity of infection at peripheries and rural areas. Detailed history and CBC was performed for all the cases. Different ratios and indexes such as systemic inflammatory index (SII), Neutrophil lymphocyte ratio (NLR), platelet lymphocyte ratio (PLR) were assessed. A total of 862 cases with a mean age of 49.9 ±17.4 years were enrolled. Hemoglobin level, lymphocyte (count per liter and percentage) were significantly lower in patients admitted in ICU as compared to patients admitted in the isolation ward and private ward (p <0.05). However, TLC, neutrophils, platelet count were higher in patients admitted to ICU (p <0.05). The Various ratios such as SII, NLR, and PLR showed significantly higher value in cases admitted in ICU (p <0.05). The TLC, neutrophil count, neutrophil percentage, SII, NLR, and PLR were significant predictors of severe disease (admission in ICU) with high diagnostic accuracy. We show that complete blood count method is a simple, readily available, rapid, and inexpensive tool that can be utilized for diagnosis and can predicting the severity of COVID 19 where RTPCR or trained staff is not available. Thus, NLR (%), SII, PLR, and TLC can predict severe illness with high accuracy.

## Background:

Corona virus, an infectious viral disease has been declared pandemic in March 2020 [1]. This is caused by the RNA virus (SARS CoV) in the family Coronaviridae [2,3]. The first case of COVID 19 infection was reported from Thrissur, Kerala, India, on 27th January 2020 [4]. The spectrum may range from mild respiratory illness (including asymptomatic cases) to severe systemic disease, including gastrointestinal, cardiovascular, neurological, immunological, and hematopoietic systems [5,6].

Literature suggests that the severity of infection is associated with elderly age, comorbid conditions such as obesity, diabetes, hypertension, chronic respiratory disease etc. [7,8]. Early diagnosis and treatment play a significant role in managing infection, as the mortality rate may be high, especially in severe cases. In extreme cases, mortality can be observed within a short duration of onset [9]. The COVID profile includes assessment of hematological parameters such as CBC, including total and differential leukocyte count, CRP, Serum ferritin, LDH, procalcitonin, D-Dimer, etc [10]. The role of neutrophils, lymphocytes, and platelets is evident in the inflammatory process. While themselves may use these parameters as inflammatory markers, their ratios to one another may also be indicators of early infections [11-13]. Though the role of d-dimer, C-reactive protein, Ferritin, and coagulation profile in predicting the severity of the disease has been established in previous studies [14]. These tests are costly not be conducted in all the cases. Therefore, it is of interest to compare the hematological profile (using Complete blood count) of COVID patients admitted in ICU, private ward, and isolation ward with varying severity to help predict the disease.

## Methodology:

This study was conducted as an observational study in the Department of Pathology, Dedicated COVID hospital of Government Medical College Ratlam (M.P) for a period of 12 months (from 1st July 2020 to 30th June 2021). Testing COVID at our study center was done by RT-PCR using QIAGEN and Thermofischer instrument in the microbiology department. All the confirmed cases of COVID 19 by RTPCR, with either at least one sign or symptom of either fever or acute respiratory disease (cough and respiratory distress); belonging to the age range of more than 12 years; admitted in ICU, HDU, ward of our center during the study period were included in the study. However, patients negative with RTPCR or RAT and asymptomatic cases were excluded from the study.

The Approval from our institutional ethics committee was taken with reference no. GMC RATLAM/IEC/2020/P-06 on 30/06/2020. All the patients fulfilling inclusion criteria were enrolled. Detailed sociodemographic and clinical history was obtained from all the study participants using a proforma. Blood sample for CBC was studied in the central clinical laboratory using Swalab alfa plus 3 part hematology analyzer.CBC parameters included in our study were total leucocyte count, differential leukocyte count (neutrophils, lymphocytes), platelet count, mean platelet volume. In the present study, we observed different ratios and indexes such as systemic inflammatory index (SII), Neutrophil lymphocyte ratio (NLR), Platelet lymphocyte ratio (PLR). Systemic inflammatory index (SII) was obtained using the following formula- thrombocyte count × neutrophil count/lymphocyte count [15].

##  Statistical analysis:

Data was compiled using MsExcel and analyzed using IBM SPSS software version 20. Categorical data expressed as frequency and percentage, whereas numerical data were expressed as mean and standard deviation. One-way ANOVA was used to compare the hematological parameters among patients admitted in ICU, private, and isolation wards. Sub-group analysis is done using Tukey HSD. Chi-square was used to assess the association of gender with a place of admission. A P-value less than 0.05 considered statistically significant.

## Results:

**Figure 1:**
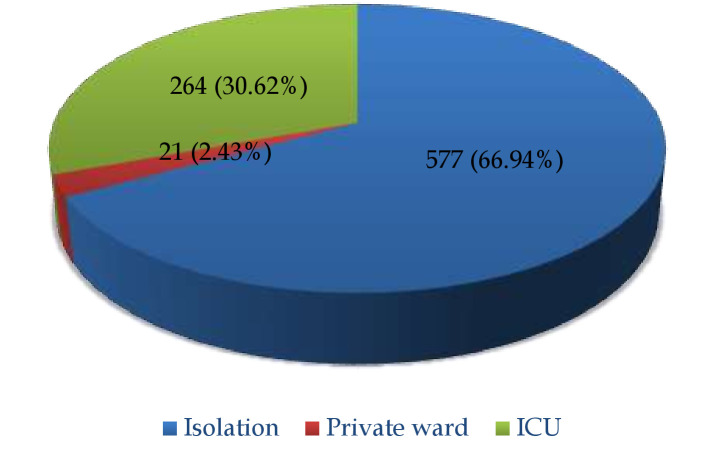
Distribution of place of admission

**Figure 2:**
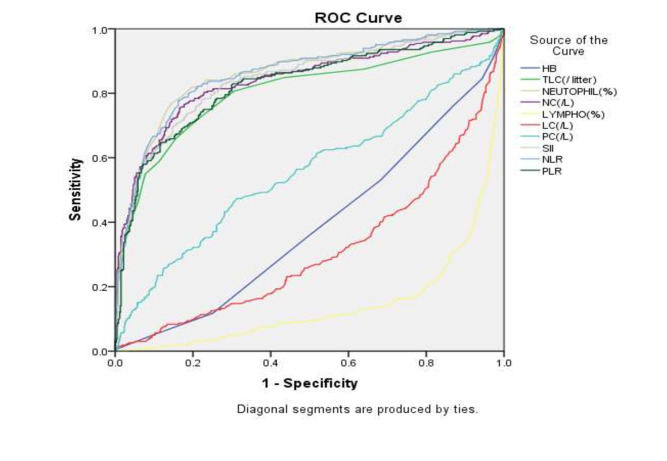
ROC Curve to determine the area under the curve for various haematological parameters

A total of 862 cases fulfilling the inclusion criteria were enrolled in our study. The mean age of patients was 49.9 ± 17.4 years. About 64.9% of cases were males, whereas the remaining 35.1% of cases were females. The majority, i.e., 66.9%, cases were admitted in the isolation ward, whereas 30.6% of patients were admitted in ICU (Figure 1).  In the present study, the mean age of patients admitted in ICU was significantly higher (59.3±14.8 years) as compared to those revealed in the private ward and isolation wards (p<0.05). Similarly, male gender was significantly associated with higher admissions in ICU (64.8%) and isolation (66%) as compared to private wards (p<0.05) (Table 1 - see PDF). Our study revealed that all the parameters used in CBC were a significant predictor of severity. Hemoglobin level, lymphocyte (count per liter and percentage) was significantly lower in patients admitted in ICU as compared to patients admitted in isolation and private ward (p<0.05). However, TLC, neutrophils, platelet count were higher in patients admitted to ICU (p<0.05). In addition, various ratios such as SII, NLR, and PLR showed significantly higher value in cases admitted in ICU (p<0.05) (Table 2 - see PDF). The above Table 3 - see PDF revealed a statistically significant difference in mean hemoglobin level between isolation and private ward and ICU and isolation (p<0.05). The mean difference in all the parameters was statistically significant between ICU and isolation ward patients (p<0.05). However, all the hematological parameters showed a statistically significant difference between patients admitted in ICU and private ward except hemoglobin (p<0.05) (Table 3 - see PDF). Table 4 (see PDF) and Figure 2 shows ROC curve analysis of various hematological parameters for predicting ICU admission. The parameters with AUC>0.8 and a p-value of less than 0.05 were considered as having higher precision. Thus, TLC, neutrophil count, neutrophil percentage, SII, NLR, and PLR were significant predictors of severe disease (admission in ICU) with high diagnostic accuracy.

## Discussion:

The COVID -19 pandemic initiated from Wuhan, China, rapidly spread across all the countries all over the World. As the virus is highly infectious and the mortality rate is also high, early diagnosis is essential in the management of COVID19 infected cases. RTPCR is the sensitive test for the diagnosis of COVID 19. However, increased caseload and a limited number of trained staff lead to delay in reporting of cases. Hence, each parameter that helps in the early diagnosis of infection can be utilized. CBC is one such parameter that is simple, cost-effective, easily accessible, and can be done even in the peripheral institute [16]. Our study was therefore conducted at a tertiary care center to determine the diagnostic accuracy of CBC parameters and specific ratios derived from CBC such as NLR, PLR, and SII. A total of 862 cases with a mean age of 49.9±17.4 years were enrolled in our study. The male predominance of COVID cases was observed in our study with a male: female ratio of 1.82:1. Based upon the severity of infections, patients were admitted to ICU, isolation, and private wards. Isolation and private ward patients were suffering from a mild illness. Similarly, the mean age of COVID-infected patients in a study by Usul et al. was 46.2 ± 15.5 years, and about 69.3% were males [16] Guan et al. documented the median age of patients as 47 years, and about 52.1% were males [17].

Our study documented a significant association of age with the severity of infection, i.e., the mean age of patients admitted in ICU was significantly higher (p<0.05). Advanced age and male gender have been associated in previous studies by Mahase et al. [18] and Yang et al. [19]. This has been attributed to associated co morbid conditions among the elderly, robust immune response as compared to young patients, and high risk of developing ARDS among them, which is the fundamental patho physiology of COVID [20]. Also, there may occur a prolonged pro inflammatory response secondary to age-related T and B cell dysfunction [21].

Amongst various hematological parameters, we documented a significantly lower level of hemoglobin in patients admitted in ICU as compared to those permitted in the ward. Though total leucocyte count, as well as neutrophil count, increased significantly with an increase in severity, lymphocytopenia was marked in severe disease. Platelet counts were also significantly higher in patients admitted to ICU (p<0.05). We observed a significant difference in all the parameters between patients admitted in ICU and isolation ward (p<0.05). Between patients admitted in ICU and private ward, all the hematological parameters showed statistically significant difference except hemoglobin. We also aimed at assessing the utility of specific ratios such as SII, NLR, and PLR, which derived from CBC. SII, a prognostic biomarker of sepsis, comprises three parameters, i.e., platelet, neutrophils, and lymphocyte. All these parameters may reflect the balance between the host immune system and inflammatory status [22]. Though its utility has been established in the prognosis of cancer, recently, its role in the diagnosis of COVID 19 has been suggested [16]. Due to marked lymphocytopenia in cases with severe infection admitted in ICU, raised SII, NLR and PLR were found in these patients. Our study findings were supported by the results of Singh et al., in which the authors documented significantly greater lymphocytopenia in patients admitted in ICU as compared to those admitted inward and HDU [23].

Lymphocytopenia is a characteristic feature in viral disease, which has been attributed to cytopathic effect due to affinity of virus for ACE inhibitors of lymphocytes [24]. Another possible explanation associated with lymphocytopenia includes exaggerated inflammatory response leading to granulopoiesis and lymphocyte apoptosis [25]. Significantly raised NLR observed in patients with ICU indicates the patient's immune response. Stress-induced neutrophils reaction along with granulopoiesis leads to an increase in neutrophil count rising in response to stress, which, when overwhelming, induces lymphocytes [23]. Exaggerated secretion of neutrophils may lead to organ damage, development of ARDS, and death as a feature of Neutrophil Extracellular Traps (NETs) [26]. Neutrophil infiltration has also been observed in pulmonary capillaries in certain patients on autopsy, which may be associated with mortality [27]. The area under the curve analysis and receiver-operating curve was done to assess the diagnostic accuracy of these hematological parameters. Area under the curve was maximum for Neutrophil % (AUC-0.861; 95% CI-0.807-0.871) followed by NLR (AUC- 0.860, 95% CI- 0,832-0.889) and SII (AUC-0.841, 95% CI- 0.81-0.872). TLC, neutrophil count per liter, as well as PLR had AUC >0.80. These parameters can be utilized to diagnose COVID 19 infection, especially at the remote and peripheral areas where only CBC can be done. Similarly, Usul et al. documented the area under the curve to be maximum for neutrophils, followed by lymphocyte and NLR (>.80; p<0.05) [16]. Yang et al., however, documented the highest AUC for NLR (0.743) at the cutoff value of 3.3 with the specificity of 63.6% and specificity of 88%. [19] The AUC for NLR was 0.88 in a study by Sun et al. for the diagnosis of COVID [28]. Though NLR has maximum AUC, in our study, neutrophil% followed by NLR had maximum AUC. The study had certain limitations, only positive symptomatic cases were included, and thus the diagnostic accuracy was assessed for predicting the severity of the disease. In addition, the sample size was small, and the effect of comorbidities, as well as the effect of age with severity, could not be assessed.

## Conclusion:

The complete blood count is a simple, readily available, rapid, and inexpensive tool that can be utilized for diagnosis and predicting the severity of COVID 19 where either RTPCR or trained staff is not available. Neurophil %, NLR, SII, PLR, and TLC, can predict severe illness with high accuracy.
